# Atrophic Myenteric and Submucosal Neurons Are Observed in Parkinson's Disease

**DOI:** 10.1155/2019/7935820

**Published:** 2019-06-19

**Authors:** Bodil Ohlsson, Elisabet Englund

**Affiliations:** ^1^Lund University, Skane University Hospital, Department of Internal Medicine, Malmö, Sweden; ^2^Lund University, Skane University Hospital, Division of Oncology and Pathology, Lund, Sweden

## Abstract

**Aim:**

Parkinson's disease is often accompanied by gastrointestinal symptoms, especially constipation. Microscopic studies of the enteric nervous system and enteric neuropathy have often been performed by immunostaining in the myenteric plexa. The aim of the present study was to examine whether pathologic changes could be identified by conventional hematoxylin and eosin (H&E) staining and could also be seen in the submucosal plexa.

**Materials and Methods:**

In 20 deceased cases (11 male/9 female) of Parkinson's disease, the intestinal tract was investigated for potential neuroganglionic disease. Ten cases (7 male/3 female) of non-Parkinson, intestinally asymptomatic individuals were used as controls. Specimens from the jejunum and colon were sampled. The material was treated with standard histopathological procedures, i.e., fixed in formaldehyde solution, dehydrated and embedded in paraffin, sectioned at 5 *μ*m thickness, and stained with H&E and immunostaining for *α*-synuclein.

**Results:**

In 15 cases (7 male/8 female) of Parkinson's disease, atrophic/pycnotic nerve plexus cells were present, i.e., signs of ganglionic degeneration in the submucosal and/or myenteric plexa, mostly identified in both loci, by H&E staining. In some cases, the degenerative signs were mild, however, corroborated by findings of *α*-synuclein deposits in the ganglion cells. The remaining 5 cases showed no signs of degeneration in the H&E staining, but immunostaining revealed minimal *α*-synuclein deposits in 3 cases. None of the controls showed any ganglionic degeneration/α-synuclein deposits.

**Conclusion:**

It seems possible to identify a morphologic intestinal disease substrate in Parkinson's disease by H&E staining, showing ganglion cell pycnosis and degeneration in both plexa. This finding may indicate a potential to diagnose enteric neuropathy in highly accessible sites.

## 1. Introduction

The myenteric plexus regulating gastrointestinal (GI) motility and the submucosal plexus regulating GI secretion constitute the enteric nervous system (ENS), which is a part of the autonomous nervous system (ANS). So far, in most studies concerning ENS and enteric neuropathy, the myenteric plexus has been examined since it is supposed that the submucosal plexus is less vulnerable to damage and often unaffected when examined [1, 2]. Nevertheless, neuropathic changes have been described in the submucosal plexus in patients with functional dyspepsia [3], inflammatory bowel disease [4], colonic inertia [5, 6], and intestinal pseudo-obstruction [7]. Traditional studies of ENS is performed using immunocytochemistry of several proteins [1–3, 5], which make the studies time-consuming to evaluate, and only a few laboratories in the world have the ability to accomplish this for clinical use. Furthermore, the methodology to study myenteric plexus demands full-thickness biopsies. With a high amount of GI symptoms in the population in patients of all ages, it is of great importance to improve and focus on how to diagnose enteric neuropathy as an etiology to symptoms in various diseases. A simpler diagnostic method on the more accessible submucosal plexus is warranted.

Parkinson's disease, which is characterized by degeneration of dopaminergic neurons in the substantia nigra and the nigrostriatal circuits, often presents itself with GI symptoms. The patients may experience symptoms from the entire GI tract, but especially from the colon in the form of constipation [8]. Analyses of GI samples have revealed a high prevalence of Lewy bodies and deposition of *α*-synuclein at an early phase of the disease [9–12]. However, the sensitivity and specificity of immunohistochemistry with staining of *α*-synuclein in Parkinsons's disease have been questioned [13, 14]. The few immunostaining studies on ENS in Parkinson's disease could not reveal any degeneration in the myenteric plexus or neuron loss in the submucosal plexus [12, 15].

The aim of the present pilot study was to examine whether conventional staining with hematoxylin and eosin (H&E) alone could be used to diagnose enteric neuropathy in GI biopsies, even if additional immunohistochemistry may also be used.

Furthermore, we wanted to evaluate both the myenteric and submucosal plexa in Parkinson's disease, compared to GI biopsies in healthy controls.

## 2. Materials and Methods

The study was performed according to the Declaration of Helsinki at Skane University Hospital, Lund, Sweden. No individual patient identification was involved, why no ethical approval was necessary.

### 2.1. Tissue Sampling

Full-thickness specimens from the jejunum (mean thickness 1.2 mm) and colon (mean thickness 1.7 mm) were sampled at autopsy in 20 deceased cases of Parkinson's disease (11 male/9 female), with median age 77 (73–84) years, who fulfilled the criteria of Parkinson's disease, i.e., parkinsonism with bradykinesia in combination with rest tremor and/or rigidity and supportive criteria, in the absence of exclusion criteria and red flags [16].

The Parkinson cases were not consecutive in this first investigation but were incorporated in the study according to availability of tissue sampled at autopsy. The deceased patients were referred for autopsy at the Department of Pathology for different reasons—mostly with the request for investigation of final cause of death—sometimes with request also for investigation of the brain disease. Autopsies were primarily performed by different pathologists; hence, the sampling was not specifically aimed at collecting for this project, and tissue amount and local origin varied between the cases. Corresponding samples were collected from 10 age-matched cases from non-Parkinson, intestinally asymptomatic individuals (7 male/3 female), with median age 78 (62–83) years, which served as controls. The intestinal tract was investigated for potential neuroganglionic disease.

The material was treated with standard histopathological procedures, i.e., fixed in formaldehyde solution and dehydrated and embedded in paraffin. The samples were then sectioned at 5 *μ*m thickness and stained with H&E. There was no serial sectioning performed, but one of the authors (E. E.) made ascertain that ganglion cells were visible in all specimens.

For immunohistochemistry to detect *α*-synuclein, antigen retrieval was performed, followed by incubation with quenching solution to block activity of endogenous peroxidase. Sections were incubated with primary monoclonal mouse IgG antibodies overnight (LB-509, Zymed Laboratories, San Francisco, CA, USA, dilution 1 : 1000). Applied biotin-conjugated secondary antibodies were then added (Dilution 1 : 200, Vector Laboratories, Burlingame, CA, USA), followed by washing and incubation with ABC solution (PK-6100, Vector Laboratories) and then DAB solution (SK-4100, Vector Laboratories).

## 3. Results

**Figure 1:**
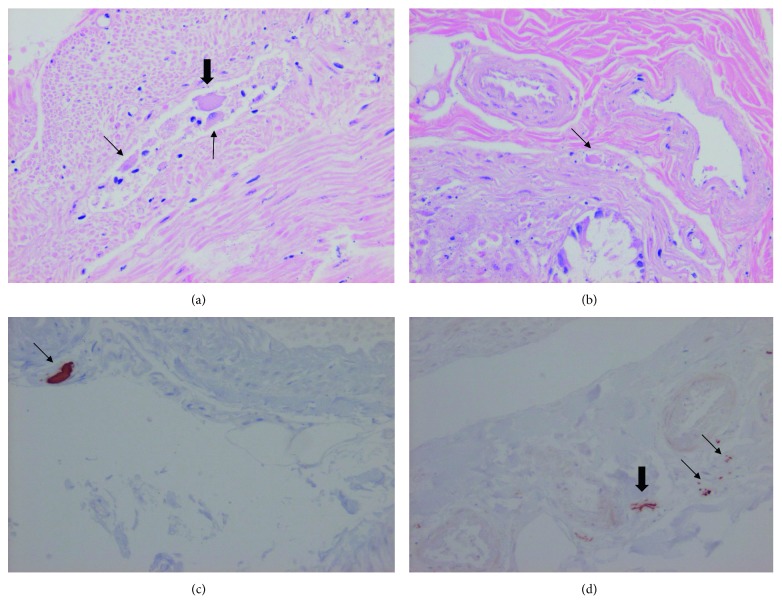
(a) Myenteric shrunken plexus with one normal-sized neuron without nucleus (thick arrow) and two small atrophic/pyknotic neurons (thin arrows) (hematoxylin and eosin staining). (b) Submucosal plexus with one single atrophic neuron (arrow) (hematoxylin and eosin staining). (c) A large *α*-synuclein immunostaining of a submucosal plexus (arrow). (d) Smaller *α*-synuclein immunostaining of two submucosal plexa (thin arrows) and one axon (thick arrow) (magnification ×200).

In 15 cases (7 male/8 female) of Parkinson's disease, there were atrophic/pycnotic nerve plexus cells, i.e., signs of ganglionic degeneration in the submucosal plexa (*n* = 10) and in the myenteric plexa (*n* = 13), mostly in both (*n* = 9), observed in H&E staining. Small, shrunken neurons were found next to normal-sized neurons without any visible nucleus in myenteric plexus (Figure 1(a)). In one submucosal plexus, only a single neuron was found (Figure 1(b)). In some cases, the degenerative signs were mild, however corroborated by findings of positive *α*-synuclein immunostaining in submucosal plexa and axons (Figures 1(c) and 1(d)). In the remaining 5 cases, there were no signs of degeneration in the H&E staining, but immunostaining revealed minimal *α*-synuclein deposits in 3 of these cases. None of the controls showed any signs of ganglionic degeneration and the *α*-synuclein staining indicated no positivity.

## 4. Discussion

The main finding in the present study was that atrophic/pycnotic neurons were visualized in Parkinson's disease in conventional H&E staining, thus without using immunohistochemistry staining. Since the degenerative changes were accompanied by *α*-synuclein depositions and these depositions were present in some samples without any neuron degeneration, it is reasonable to believe that the protein depositions appear prior to the degeneration.

The pathophysiology behind the GI symptoms in Parkinson's disease is unknown. Lewy bodies and Lewy neurites with aggregation of the presynaptic protein *α*-synuclein are the pathological hallmarks of Parkinson's disease, which may be found in different parts of the nervous systems [17]. In the brain, the pathological process progresses in a predetermined sequence [18]. Depositions of *α*-synuclein have previously been described at an early stage in both myenteric and submucosal plexa [9, 10, 12]. Although the sensitivity and specificity of *α*-synuclein deposits in Parkinson's disease has been questioned [13, 14], *α*-synuclein is found in much higher proportion of biopsies from patients with Parkinson's disease than from healthy controls [19]. It is uncertain whether the disease starts in the central nervous system (CSN) or ENS since there are strong correlations between GI symptoms, motor symptoms, and autonomic dysfunction in the disease [8] and constipation may be present several years prior to motor symptoms [20].

Although no overall loss of submucosal neurons could be found in Parkinson's disease, a reduced number of submucosal mRNA coding for vasoactive intestinal peptide (VIP) and its receptors and neurons containing (VIP) was described, suggesting that the total number of VIP-containing neurons was decreased [21]. VIP is a central neuropeptide in the gut and constitutes a major subpopulation in the submucosal plexus, where it stimulates intestinal secretion [22, 23]. In the myenteric plexus, VIP acts directly on smooth muscle cells as an inhibitory transmitter. Furthermore, VIP is considered a neuroprotective peptide with anti-inflammatory properties [24], which may be important since infiltration of inflammatory cells are correlated with changes in calcium transient amplitudes [3]. Altogether, a reduced number of VIP-containing neurons may be of importance for development of GI symptoms. No associations could be found between constipation and central or peripheral dopaminergic pathology [12, 25] or myenteric histopathological parameters [12]. However, constipation in Parkinson's disease may be caused by decreased intestinal secretion due to damage in the submucosal plexa and does not necessarily involve myenteric or dopaminergic neurons.

Swollen, vacuolated, or chromatolytic ganglion cell cytoplasm with lipofuscin granules have previously been described as a rare finding in Parkinson's disease, but not further discussed [9, 10]. The question remains why submucosal neural changes observed in the present study have not been discussed previously. First, it may depend on a greater interest among scientists for the myenteric plexus, which is assumed to be the most important plexus for GI function. Second, improved technical instrumentation with enhanced possibility to discover changes may be another explanation. Third, all studies are performed in series with low numbers of cases. Different degrees of disease severity among the few cases may explain various findings. A prior study in functional dyspepsia did not reveal any neuropathy in conventional histopathological staining; only in immunohistochemistry staining could decreased numbers of neurons be demonstrated [5]. However, massive submucosal ganglia and hyperplasia of submucosal plexus, without degenerative changes, could be seen in H&E staining in intestinal neuronal dysplasia type B and colonic inertia [6, 26]. Fourth, patchy changes present only at some sites may be missed in a single full-thickness biopsy. According to Wakabayashi et al. [9], the Lewy bodies were most often observed in the submucosal plexus of the lower esophagus, a site seldom biopsied to study the ENS.

The limitation of the present pilot study is the retrospective character with biopsies from deceased subjects. These patients were followed by the Department of Neurology, and the amount or degree of GI symptoms were not estimated in any systematic way. Thus, we could not have correlated our pathological findings with clinical features of dysmotility, even if we had had the identification numbers of the patients. On the other hand, a strength is that we had access to full-thickness biopsies and to well-selected control case biopsies, hence enabling a search for both submucosal and myenteric ganglion cells.

In summary, extracerebral neuron degeneration in Parkinson's disease can be identified in myenteric plexa as well as in submucosal plexa by conventional H&E staining. In the future, collaborations between scientists focused on the CNS, ANS, ENS, and peripheral nervous system are necessary to increase the knowledge about interactions of the different parts of the nervous systems, to better understand pathophysiology and development of symptoms. It is necessary to use highly accessible sample sites for simple diagnosing, to improve patient care, and to develop improved targeted therapies. After description of neurodegenerative changes in the submucosal plexa, prospective studies combining questionnaires enquiring GI symptoms and endoscopic sampling can be combined to examine the correlation between symptoms of GI dysmotility and signs of GI histopathology.

## Data Availability

Original data can be obtained upon request to the corresponding author.
